# Orientational control of molecular scale thermoelectricity†

**DOI:** 10.1039/d2na00515h

**Published:** 2022-10-07

**Authors:** Majed Alshammari, Alaa A. Al-Jobory, Turki Alotaibi, Colin J. Lambert, Ali Ismael

**Affiliations:** Physics Department, Lancaster University Lancaster LA1 4YB UK k.ismael@lancaster.ac.uk; Department of Physics, College of Science, Jouf University Sakaka Saudi Arabia; Department of Physics, College of Science, University of Anbar Anbar Iraq; Department of Physics, College of Education for Pure Science, Tikrit University Tikrit Iraq

## Abstract

Through a comprehensive theoretical study, we demonstrate that single-molecule junctions formed from asymmetric molecules with different terminal groups can exhibit Seebeck coefficients, whose sign depends on the orientation of the molecule within the junction. Three anthracene-based molecules are studied, one of which exhibits this bi-thermoelectric behaviour, due to the presence of a thioacetate terminal group at one end and a pyridyl terminal group at the other. A pre-requisite for obtaining this behaviour is the use of junction electrodes formed from different materials. In our case, we use gold as the bottom electrode and graphene-coated gold as the top electrode. This demonstration of bi-thermoelecricity means that if molecules with alternating orientations can be deposited on a substrate, then they form a basis for boosting the thermovoltage in molecular-scale thermoelectric energy generators (TEGs).

## Abstract 2

Through a comprehensive study, we demonstrate that single-molecule junctions formed from asymmetric molecules with different terminal groups can exhibit Seebeck coefficients, whose sign depends on the orientation of the molecule within the junction.

## Graphical abstract

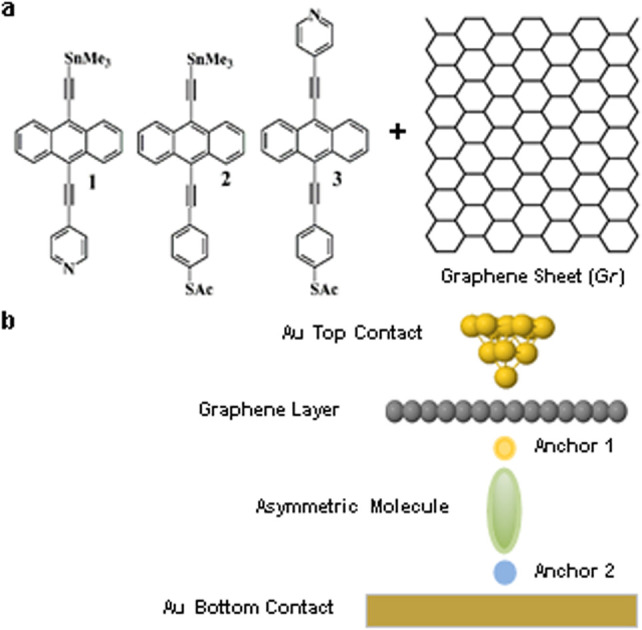

## Introduction

The desirability of converting low-grade waste heat^1–3^ into electricity has led to recent studies of the thermoelectric properties of organic polymers such as thiophenes and perylene diimides.^4–6^ However, understanding the fundamentals of their thermoelectric behaviour is complicated by difficulties in characterising their structure at a molecular scale.^7^ Single-molecule junctions and self-assembled monolayers (SAMs) have the potential to overcome this difficulty, since they are well characterised and formed from molecules of a well-defined atomic structure.^8–11^ Furthermore, their electronic and thermal transport properties can be tuned by varying their redox state, their anchor groups to electrodes and taking advantage of quantum interference (QI) effects.^12–26^

**Fig. 1:**
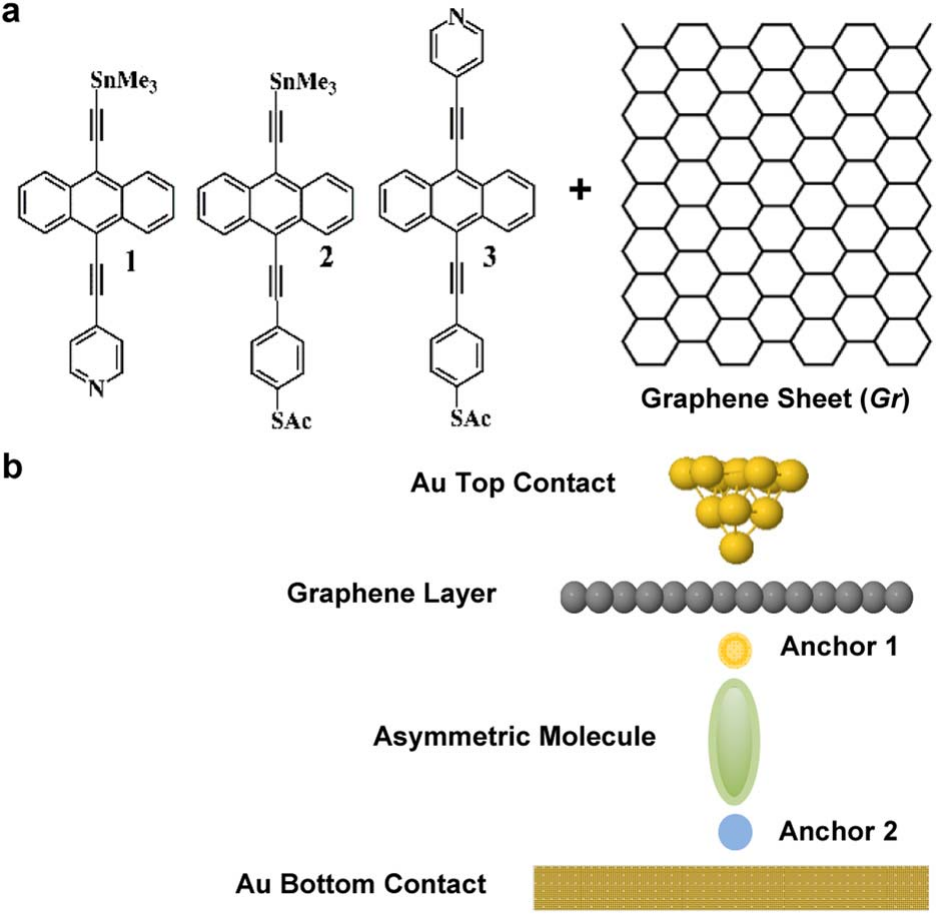
(a) Chemical structures of studied molecules 1–3, plus a graphene layer Gr. (b) Typical schematic of a fabricated junction.

High quality monolayers can be formed between symmetric^27,28^ or asymmetric electrodes.^29^ and furthermore, single-molecule QI effects^30^ can be translated to large area SAMs.^31–34^ This means that design principles developed from studies of single-molecule junctions can be utilised in more device-applicable thin film arrays of molecules. Here, our goal is to investigate new strategies towards the formation of thermoelectrically efficient devices involving highly asymmetric electrode-linker groups. In particular, we investigate whether or not the sign of the Seebeck coefficient of such junctions is sensitive to the orientation of the molecules. To explore this possibility, we studied the single-molecule junctions shown in Fig. 1b, composed of a molecule bound to a bottom flat gold electrode and to a top graphene sheet (Gr), which is in turn contacted by a gold top contact, in the form of an STM tip. In what follows, we study transport through such single-molecule junctions, formed using either of the anthracene-based molecules 1, 2 and 3. Our aim is to determine if flipping the molecules within the junction (*i.e.*, rotating them about a horizontal axis through 180°) causes the sign of the Seebeck coefficient to change. The ability of a given molecule to exhibit Seebeck coefficients of either sign is known as bi-thermoelectricity and our aim is to determine if any of the three molecules shown in Fig. 1a is bi-thermoelectric.

## Results and discussion

The transport properties of 9 junctions were investigated using a combination of density functional theory and quantum transport theory to obtain the transmission coefficient *T*(*E*) describing electrons of energy *E* passing from the source to the drain electrodes.^35^ From this, the room-temperature electrical conductance *G* and Seebeck coefficient *S* were determined.

The three asymmetric anthracene-based molecules were terminated with pyridyl, thioacetate and SnMe_3_ anchor groups (Fig. 1a). We began by calculating the optimum binding distance *d*_Anc_h__ between the anchors and the gold contact as shown in Fig. S5–S7.† It should be noted that 2 out of 3 anchors cleave when attached to a Au contact, as follows: –SAc cleaves and ends up as a Au–S contact, similarly, –SnMe_3_ forms a Au–C direct contact (–TMS). The optimum binding distance *d*_Anc_h__ between the anchors and the graphene layer was also calculated as shown in Fig. S8–S10, for more detail see Section 2 of the ESI.†

As a first step, we investigated transport through these molecules in Au–Au junctions. Each case illustrates a unique type of transport even though all of them possess two different anchors. 1 shows LUMO-dominated (Lowest Unoccupied Molecular Orbital) transport hinting that the –Py moves the LUMO closest to the Fermi energy, whereas 2 exhibits HOMO-dominated (Highest Occupied Molecular Orbital) transport indicating that the thiol moves the HOMO closer to the Fermi energy. On the other hand, 3 exhibits mid-gap transport suggesting that –Py and –SH anchors cancel each other's opposing tendency to move the LUMO and HOMO closer to the Fermi energy. The 3 cases are shown in Fig. S11–S13, and for more detail see Section 3 of the ESI.† Since Seebeck coefficient is proportional to the slope of the logarithm of the transmission coefficient *T*(*E*), 1 and 2 have Seebeck coefficients of opposite signs and 3 has a low Seebeck coefficient.

Up to this point, we explored asymmetric molecules in gold–gold junctions. The next step is to insert a graphene layer close to the top Au-contact as shown in Fig. 1b. For experimental details about the STM measurements of such Gr-based junctions, see ref. 36.

Again, we consider 3 scenarios to establish their “flipping characteristic” as follows: scenario-a where molecule 1 flips between the Gr-layer and Au-substrate, which results into 2 orientations as shown in Fig. S17.† It should be noted that, orientation-1 experiences a cleavage at the SnMe_3_ anchor, when contacting to Au to form a direct C–Au bond. Despite the fact that the position of Fermi energy changes when the molecule flips, leading to a change in the magnitude of the Seebeck coefficient, there is no change in the sign of the Seebeck coefficient, because as shown in the top panel of Fig. S18,† the transmission curves remain LUMO dominated.

For molecule 2, the top panel of Fig. S20† demonstrates that the two orientations are HOMO dominated and again there is no change in the sign of the Seebeck coefficient upon flipping.

**Fig. 2:**
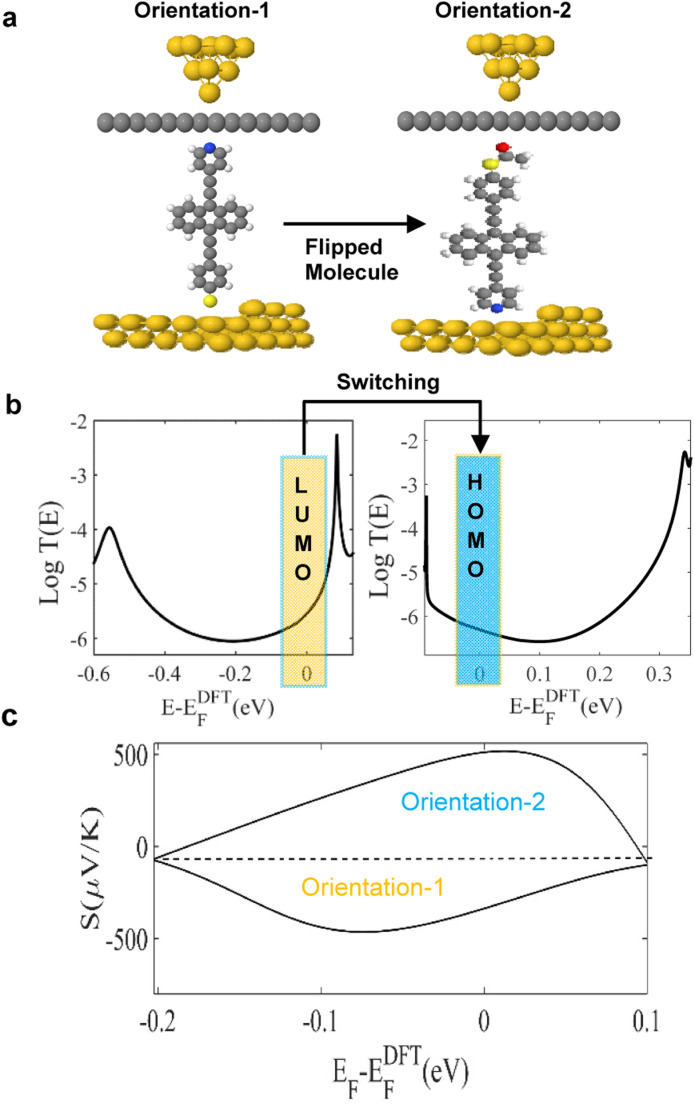
(a) Schematic illustration of molecular junctions of two orientations. Orientation-1 and -2 show how the molecule flips between the Gr layer and Au substrate. Orientation-1 is when Py linked to the Gr from one end and S to Au from the other end. Orientation-2 is the opposite, SAc linked to the Gr and Py to Au contact. (b) Zero bias transmission coefficients *T*(*E*) against electron energy *E*. The flipping characteristic switches the Fermi energy (*E* − *E*^DFT^_F_ = 0 eV), from LUMO- towards HOMO-resonance (orange and blue rectangles respectively). (c) Seebeck coefficients *S* as a function of the energy of orientation-1 and -2. Orientation-1 exhibits a negative *S*, whereas orientation-2 shows a positive *S*.

These results show that although the junctions are asymmetric, the sign of the Seebeck coefficient is insensitive to the orientations of the molecules. In contrast, as shown in Fig. 2, the sign of the Seebeck coefficient of molecule 3 is sensitive to its orientation and therefore molecule 3 exhibits bi-thermoelectricity. On the other hand, for a SAM, the measured sign of the Seebeck coefficient will be determined by the percentage of molecules adopting a given orientation. Therefore, if a SAM of 3 is deposited such that pyridyl–gold and thiol–gold bonds occur with equal probabilities, the average Seebeck coefficient of such a film will be low.

Fig. 2a illustrates the two orientations of molecule 3. This panel also shows that in orientation-1, cleavage at the thioacetate occurs, to form a Au–S contact. Panel 2b, shows the transmission coefficients *T*(*E*), of orientations-1 and -2 and demonstrates how *T*(*E*), switches from LUMO to HOMO-dominated transport (see orange and blue rectangles around Fermi level). We attribute this behaviour to the influence of the graphene layer, since in the absence of the layer, no such sign change occurs. Indeed, these results show that the top graphene coated contact defines the transport type, with Au + Gr-Py or Au + Gr-SAc, being either LUMO or HOMO dominated respectively.

## Conclusions

We have studied asymmetric systems that are capable of switching the sign and boosting the Seebeck coefficients of asymmetric single-molecule junctions. In the presence of a graphene top contact, we find that flipping the orientations of molecules 1 and 2 changes the magnitude, but not the sign of the Seebeck coefficient, whereas molecule 3 is found to be bi-thermoelectric, exhibiting Seebeck coefficients of either sign, depending on its orientation within the junction.

Based on XPS measurements of a similar junctions, sandwiching asymmetric molecules such as 1, 2 and 3, in Au + Gr-Au junctions will result into both possible orientations and will yield films with rather different Seebeck characteristics. For 1 and 2, STM measurements of single-molecule's Seebeck coefficients would fluctuate in magnitude, but not in sign, across the film. In contrast, for SAMs formed from 3, single-molecule STM-based measurements would yield values of *S*, with random signs across the film. These qualitatively distinct behaviours provide new insights into the thermoelectric properties of SAMs. They also show that in the case of 3, if the orientations of molecules in neighboring islands could be controlled, to yield SAMs with alternating orientations and therefore Seebeck coefficients of alternating signs, then these could form a basis for boosting the thermovoltage in nanoscale thermoelectric generators.

## Synthesis

For details relating to the synthesis of molecules, refer to our recent publications.^34,36^

## Author information

A. I. and C. L. conceived the concept. A. A. and M. A. co-supervised the project. A. I. and C. L. wrote the manuscript with output from all authors. M. A., A. A. and T. A. performed theoretical calculations of the flipping characteristic simulations. All authors approved the final version of the manuscript.

## Conflicts of interest

There are no conflicts to declare.

## Supplementary Material

NA-004-D2NA00515H-s001
