# Supplementary material for: Orientational control of molecular scale thermoelectricity
Source: Nanoscale Adv. 2022 Oct 7;4(21):4635–8. doi: 10.1039/d2na00515h (PMC9595198; doi:10.1039/d2na00515h)
Supplement: NA-004-D2NA00515H-s001 [file NA-004-D2NA00515H-s001.pdf]

Electronic Supplementary Material (ESI) for Nanoscale Advances.
This journal is © The Royal Society of Chemistry 2022

Supporting Information

Orientational control of Molecular scale thermoelectricity

Majed Alshammari, ‡,a Alaa A. Al-Jobory, ‡,a, Turki Alotaibi‡,a, Colin J. Lambert,[a] and Ali Ismael,[a*]

[a.] Physics Department, Lancaster University, Lancaster, LA1 4YB, UK.

Table of contents

1. Theoretical details

2. Binding energy

3.Transmission coefficient $T(E)$

4.Seebeck coefficient S

5. Flipping characteristic

6. References

1. Theoretical details

1.1 Optimised DFT Structures of Isolated Molecules

Using the density functional code SIESTA,[1, 2] the optimum geometries of the isolated molecules **1-3** were obtained by relaxing the molecules until all forces on the atoms were less than 0.01 eV / Å as shown in Fig. SI.1. A double-zeta plus polarization orbital basis set, norm-conserving pseudopotentials, with an energy cut-off of 250 Rydbergs, defined on the real space grid was used and the local density approximation (GGA) was chosen to be the exchange correlation functional.

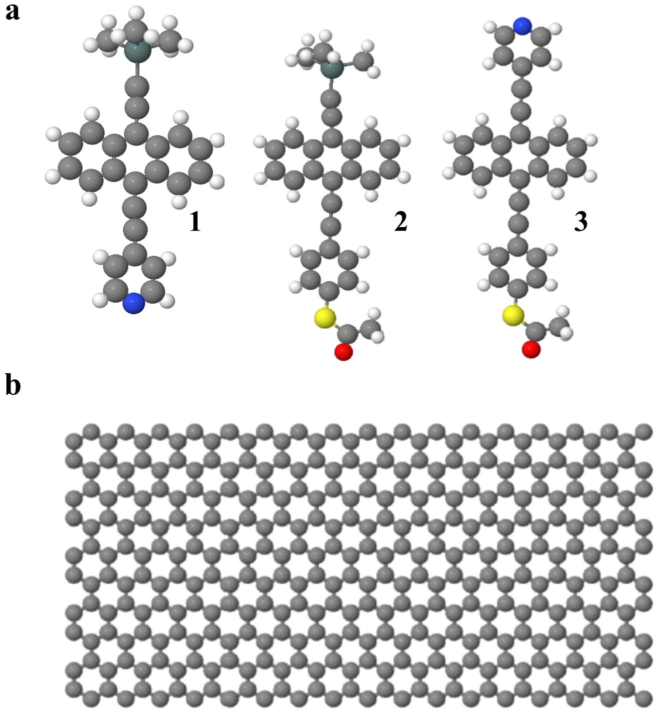

Graphene sheet (*Gr*)

Figure SI.1: a: Asymmetric anthracene-based molecules. 1: Anthracene with $SnMe_3$ and *Py* anchors, 2: Anthracene with $SnMe_3$ and *SAc* anchors, and 3: Anthracene with *Py* and *SAc* anchors. b: Graphene sheet (*Gr*)

Figure SI.1 shows three structures of asymmetric anthracene-based molecules. These structures are fully relaxed, and are as follows, 1: anthracene-based molecule with two different anchors including $SnMe_3$ and pyridine ($SnMe_3$-anthracene-*Py*), 2: anthracene-based molecule with $SnMe_3$ and thioacetate ($SnMe_3$-anthracene-*SAc*), 3: anthracene-based molecule with pyridine and thioacetate (*Py*-anthracene-*SAc*), and a graphene sheet (*Gr*). The three asymmetric anthracene-based molecules were combined with *Gr* and placed between *Au* electrodes to study their flipping features as we shall discuss later.

1.2 Frontier orbitals of the molecules.

In this section, we show the frontier orbitals of the studied molecules: highest occupied molecular orbitals (HOMO) and lowest unoccupied orbitals (LUMO), in addition to (HOMO+1), and (LUMO-1), along with their energies.

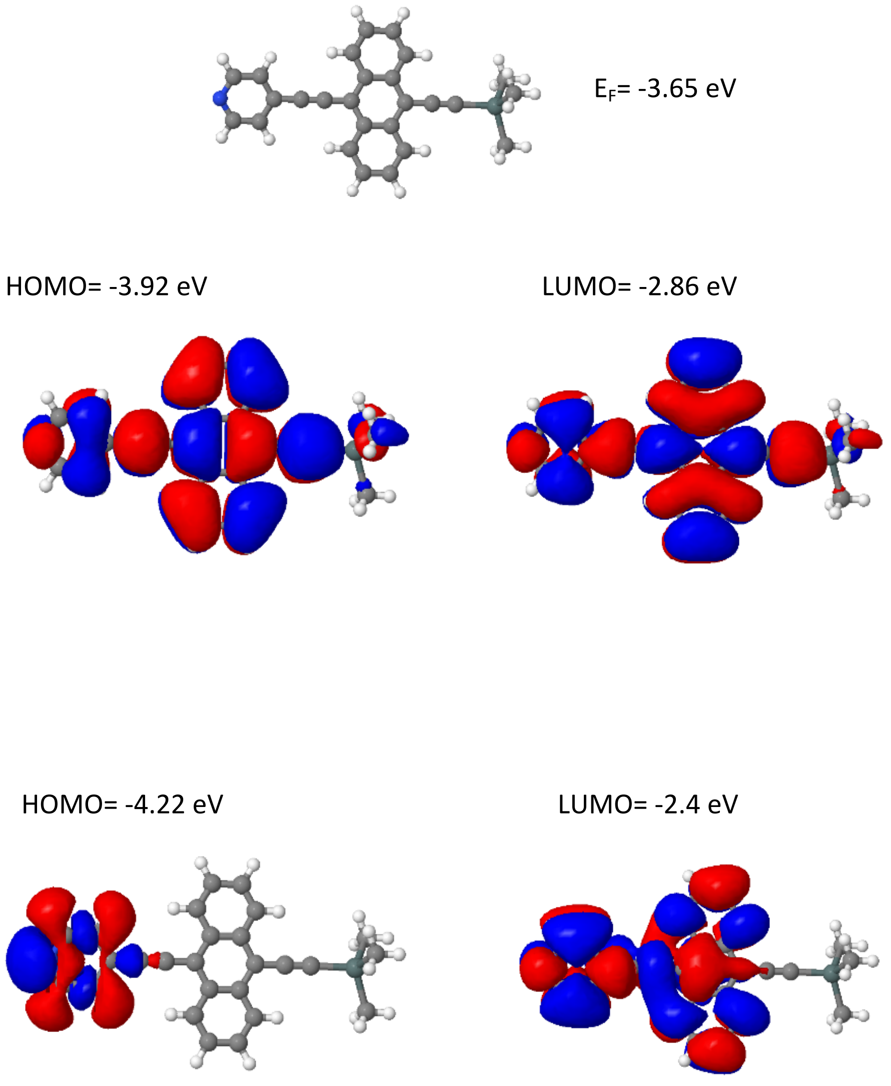

Figure SI.2: Wave function for **1**. Top panel: fully optimised geometry of **1**. Lower panel: HOMO, LUMO, HOMO-1, LUMO+1 of molecule 1 along with their energies.

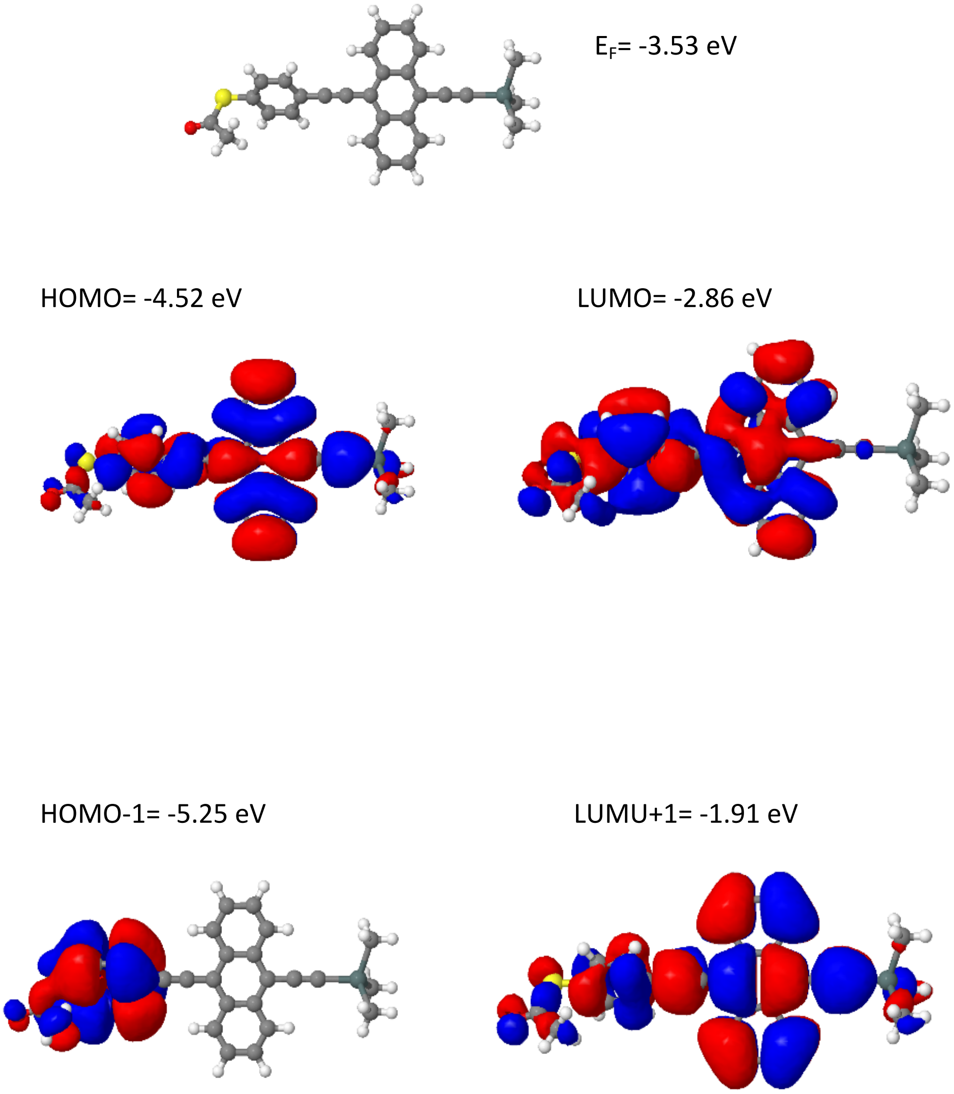

Figure SI.3: Wave function for **2**. Top panel: fully optimised geometry of **2**. Lower panel: HOMO, LUMO, HOMO-1, LUMO+1 of molecule 2 along with their energies.

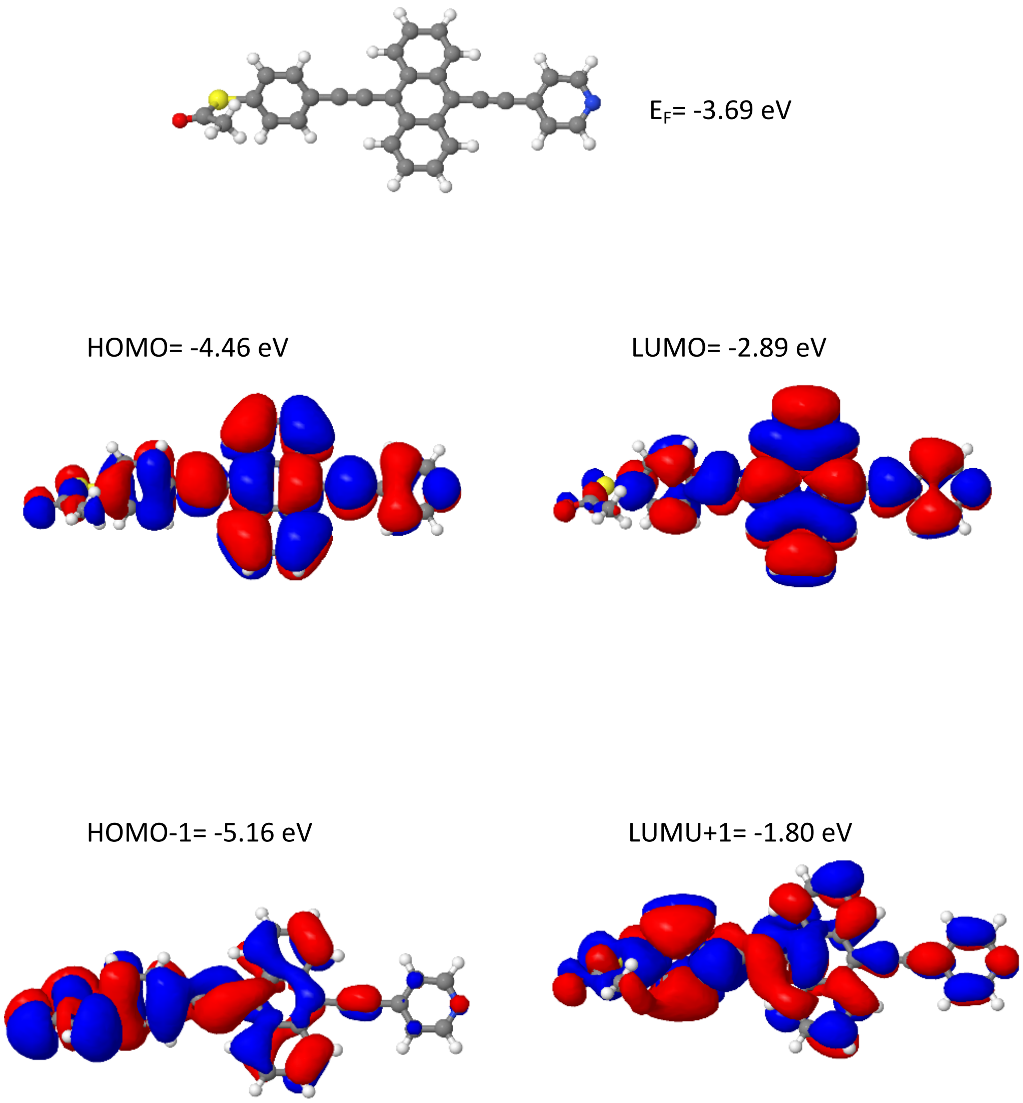

Figure SI.4: Wave function for **3**. Top panel: fully optimised geometry of **3**. Lower panel: HOMO, LUMO, HOMO-1, LUMO+1 of molecule 3 along with their energies.

2.0 Binding Energies

This section uses a combination of DFT and the counterpoise method. Briefly, the latter removes the basis set superposition errors when calculating the optimum binding distance of two objects; for more details see[3, 4] .

2.1 Binding Energy of Anthracene Core to Gold substrate:

Here, we calculated the binding energy of the anthracene-based molecules as shown in Figs. SI 5-7, on a gold substrate. These asymmetric molecules have different anchor groups including: *Py*, thiol, and

TMS (see Figs. SI 5-7). It should be noted that for both *SAc* and *SnMe3* groups of molecules **1-3** (see Fig. 1a in the manuscript), some changes occur when *SAc* and *SnMe3* groups attach to a gold metal. In particular, the *SAc* group cleaves to form a *S-Au* bond [5]. Similarly, *SnMe3* cleaves to form a direct *C-Au* bond[5].

Figure SI.5 (B1), shows that the optimum binding distance d_{Anch}. between the *Py* anchor and the *Au* to be 2.3 Å, and at approximately -0.4 eV. It is worth mentioning that in this case there the molecule remains as it is, meaning no changes as shown in Figure 1a (molecule **3**), in the manuscript.

Similarly, Figure SI.6 (B2) represents the binding energy between the thiol anchor group and the gold lead and $d_{Anch.}$ is 2.4 Å, at approximately -1.2 eV. This suggests the binding energy of the thiol anchor group is much stronger than that the *Py* anchor to *Au* electrode (compare Fig. SI.6 against Fig. SI.5). This result in agreement with the literature review, it should be noted that the *SAc* group cleaves when this group brought close to the *Au* metal to form *S-Au* bond.

Figure SI.7 (B3) exhibits the binding energy between the *TMS* anchor group and the gold lead. The TMS's (*Au-C*), binding energy lies between the *S* and *Py*, however, it is more towards the stronger binding energy (i.e. thiol) to Au with binding energy of -1 eV at $d_{Anch.}$ = 2.3 Å. These calculations suggest that both thiol and *TMS* bind to Au substrate approximately 3 times stronger than that *Py* anchor. Again, the SnMe3 group cleaves when this group brought close to the Au metal to form *C-Au* direct bond, (Note the optimum distance between the Au and Anchor labelled $d_{Anch.}$).

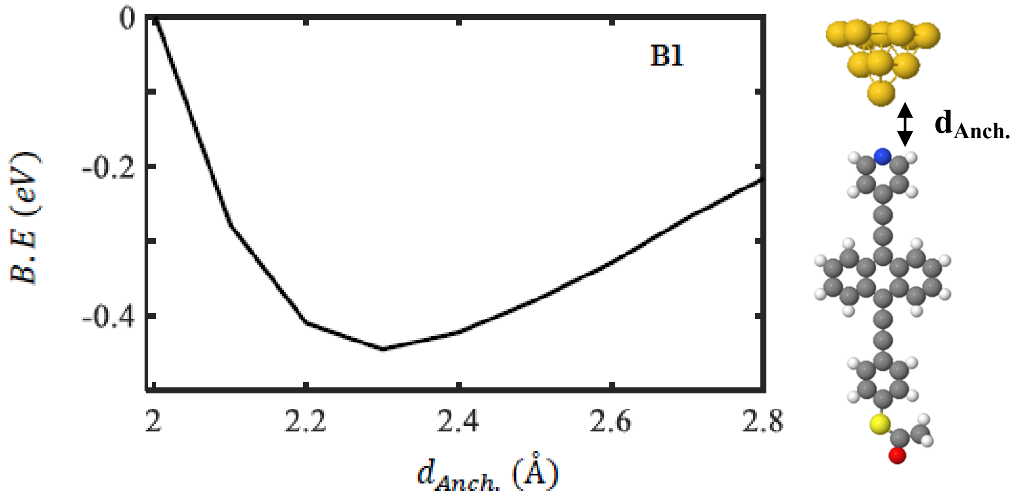

Figure SI.5: An asymmetric anthracene-based molecule configuration with thioacetate and pyridine anchors at the *Au* lead interface *Au-Py* (right). Binding energy as a function of the optimum binding distance d_{Anch}., where d_{Anch}. is found to be approximately 2.3 Å. Key: C = grey, H = white, S = light yellow, Au = dark yellow, N = blue, O = red.

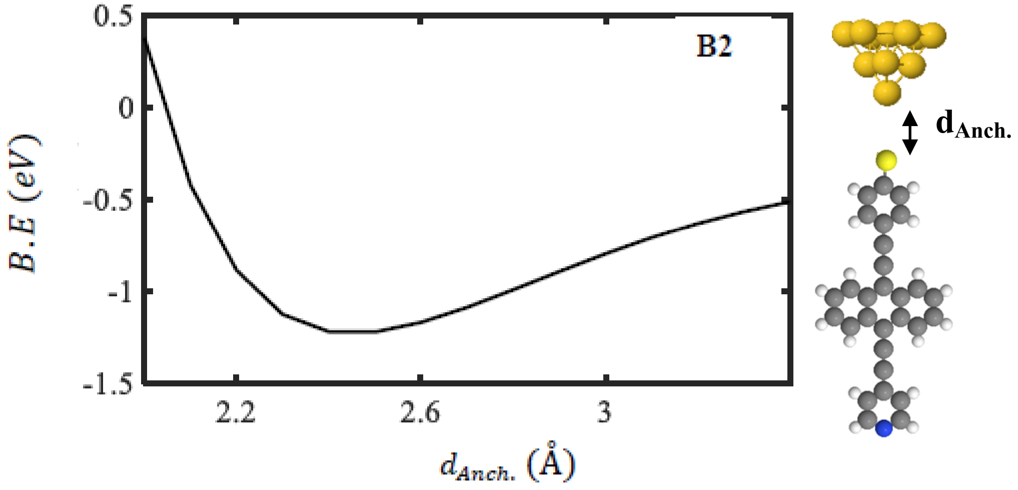

Figure SI.6: An asymmetric anthracene-based molecule configuration with thioacetate and pyridine anchors at the *Au* lead interface *Au-S* (right). Binding energy as a function of the optimum binding distance $d_{Anch.}$, where $d_{Anch.}$ is found to be approximately 2.4 Å. Key: C = grey, H = white, S = light yellow, Au = dark yellow, N = blue.

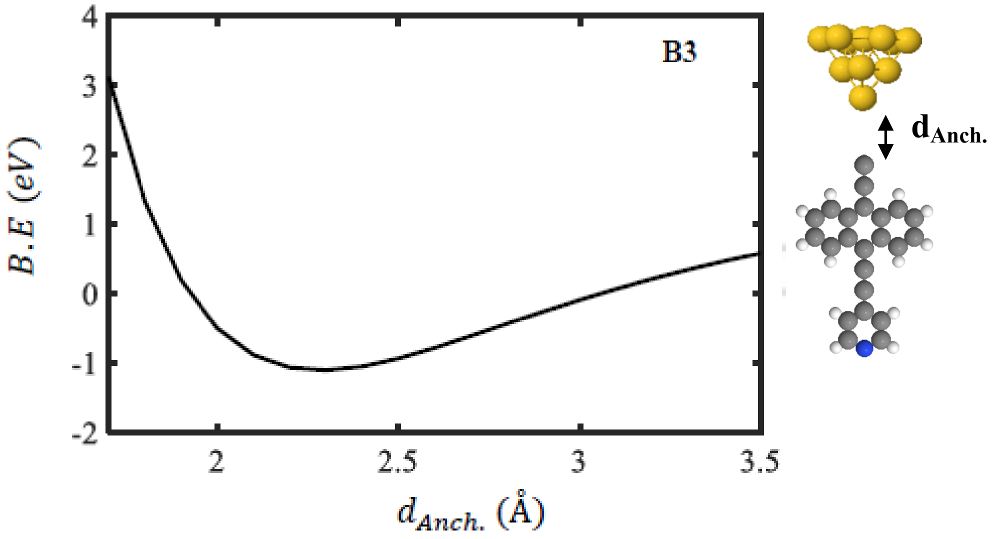

Figure SI.7: An asymmetric anthracene-based molecule configuration with thioacetate and pyridine anchors at the *Au* lead interface *Au-S* (right). Binding energy as a function of the optimum binding distance $d_{Anch.}$, where $d_{Anch.}$ is found to be approximately 2.2 Å. Key: C = grey, H = white, Au = dark yellow.

2.2 Binding Energy of Anthracene Core to Graphene sheet:

This section illustrates the second part of the binding simulations. Another three biding energies have been calculated where, we demonstrate how an asymmetric anthracene-based molecule of different anchor groups binds to a graphene sheet (*Gr*). Thus, we calculate the binding energy as a function of the optimum binding distance of a *Gr* sheet to either *Py* or $SnMe_3$ or *SAc* anchor group.

The binding energy between a *Gr* sheet and pyridine anchor is shown in Figure SI.8, where the right panel shows an asymmetric anthracene molecule linked to a graphene sheet (*Gr-Py*). The left panel represents the binding energy plot as a function of the optimum binding distance d. In this case, d is found to be approximately 3Å, and the B.E is approximately -0.14 eV. (Note the optimum distance between the graphene sheet and anchor labelled d).

Next, we connect the *Gr* sheet with an asymmetric anthracene, this time, and we calculate the binding energy, as shown in Figure SI.9 (B5). The right panel represents an asymmetric anthracene molecule linked to a graphene sheet (*Gr-SAc*). The left panel shows the binding energy as a function of the optimum distance d. and d is approximately 5.5 Å, with B.E approximately - 0.8 eV.

The last anchor to investigate with graphene sheet is *SnMe3*, we attach the *Gr* sheet to an asymmetric anthracene (*SnMe3* and *Py*), as shown in Figure SI.10 (B6). The right panel is an asymmetric anthracene molecule linked to a *Gr* sheet via *SnMe3*. In the left panel, d is found to be 5.9 Å, and the binding energy is approximately -0.6 eV.

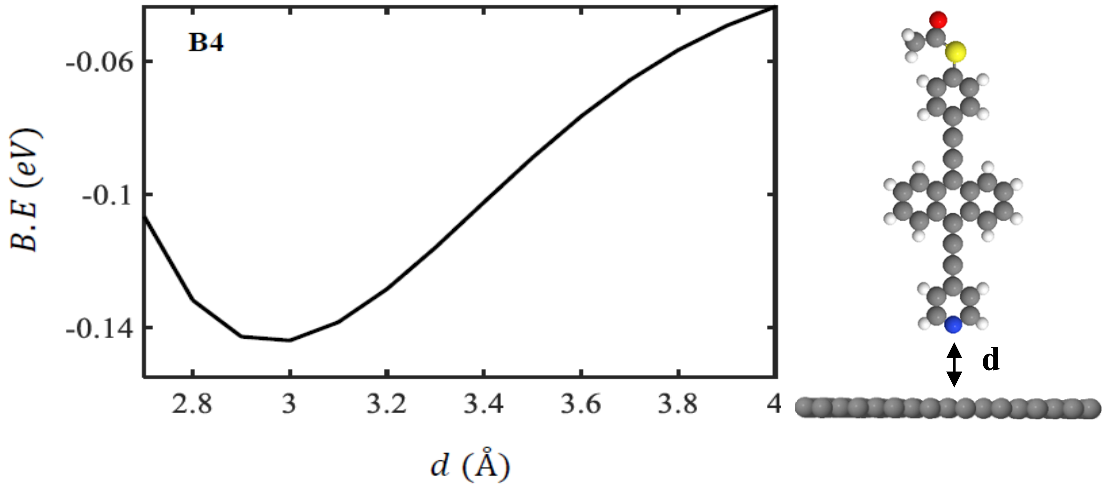

Figure SI.8: An asymmetric anthracene-based molecule configuration with thioacetate and pyridine anchors at the graphene interface *Gr-Py* (right). Binding energy as a function of the optimum binding distance d, where d is found to be approximately 3.0 Å. Key: C = grey, H = white, S = light yellow, N = blue, O = red.

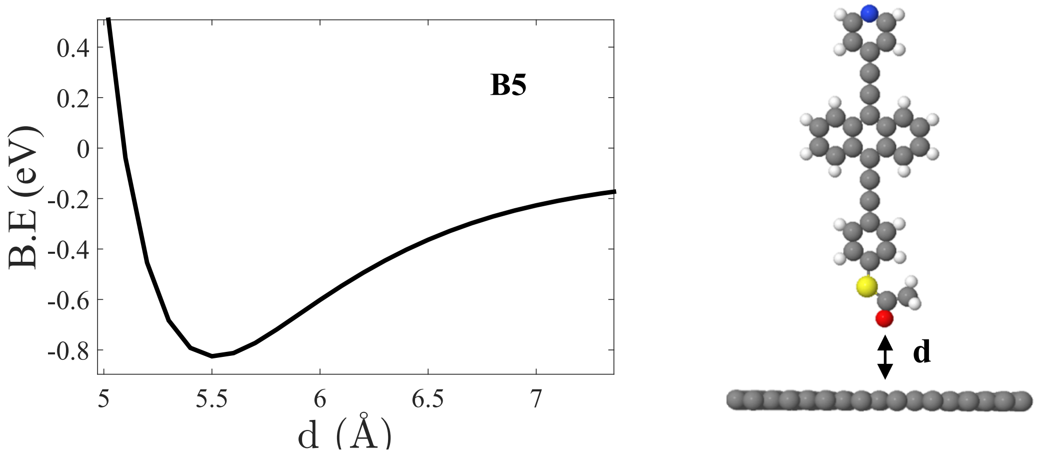

Figure SI.9: An asymmetric anthracene-based molecule configuration with thioacetate and pyridine anchors at the graphene interface *Gr-SAc* (right). Binding energy as a function of the optimum binding distance *d*, where *d* is found to be approximately 5.5 Å. Key: C = grey, H = white, S = light yellow, N = blue, O = red. B.E=-0.8 eV.

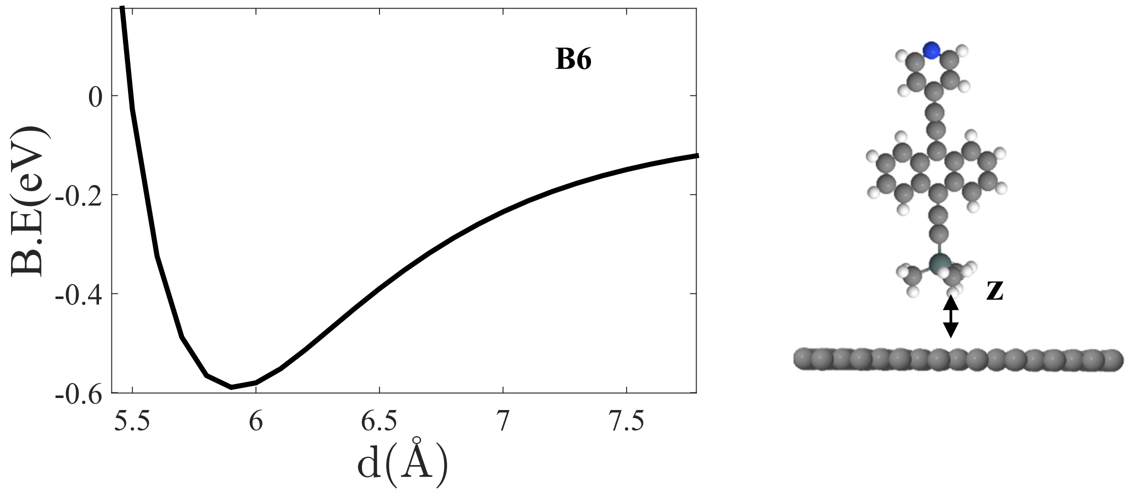

Figure SI.10: An asymmetric anthracene-based molecule configuration with thioacetate and pyridine anchors at the graphene interface *Gr-SnMe3* (right). Binding energy as a function of the optimum binding distance *d*, where *d* is found to be approximately 5.9 Å. Key: C = grey, H = white, S = light yellow, N = blue, O = red. B.E=-0.6 eV.

Table S1: Summarises all the binding energies (B.E), and optimum distances ($d_{Anch.}$ and d), calculations for three different anchor groups bind to *Au* or *Gr*.

Mol.	$d_{Anch.}$ and d (Å)	B.E (eV)
B1	2.3	-0.45
B2	2.4	-1.2
B3	2.2	-1.0
B4	3	-0.15
B5	5.5	-0.8
B6	5.9	-0.6

3.0 Transmission coefficient $T(E)$

This section investigates the transmission function of asymmetric anthracene-based core molecules with different anchor groups including *SnMe3, Py* and *SAc* for this purpose we shall explore three different cases:

3.1 Case 1: Anthracene-based of *SnMe3* and *Py* anchor groups:

Anthracene molecule with two different anchors including *SnMe3* and *Py*, has been studied as shown in Figure SI.11. If the two anchors were pyridine, one would expect this molecule to be a LUMO-dominated due to the presence of the pyridyl anchor. However, it seems the case is still true even if the molecule is asymmetric, which means two different anchors. We believe this is due to that the *Py* anchor overcomes the *TMS* (*Au-C*), even though the binding energy of *TMS* is stronger than that *Py*. It is worth mentioning that, some studies[6] demonstrate that *TMS* is a HOMO-dominated anchor and that is clearly shown in Figure SI.11, where the *TMS* pulls the DFT-predict Fermi energy ($E - E_F^{DFT} = 0$ eV) slightly away from LUMO resonance, as the pyridyl anchor is pinning the Fermi level $E - E_F^{DFT} = 0\ eV$ so close to the LUMO resonance[7, 8]. It should be noted that the SnMe3 group cleaves when it attaches to Au contact to form *Au-C* direct contact, as we discussed that above.

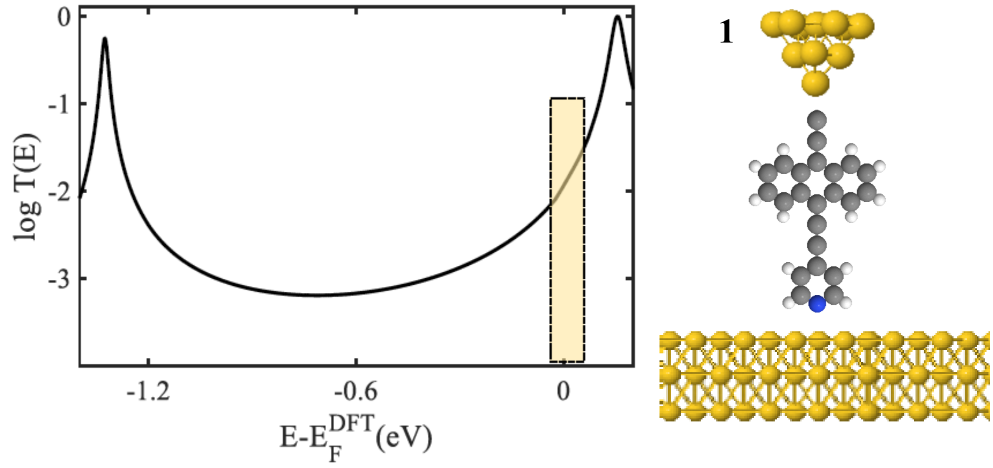

Figure SI.11: Right panel: Schematic illustrations of an asymmetric molecular junction of **1. Left panel:** Zero-bias transmission coefficient $T(E)$ of molecule **1** against electron energy E.

3.2 Case 2: Anthracene-based of *SnMe3* and *SAc* anchor groups:

In this case, we consider anthracene with two different anchors including *SnMe3* and *SAc* anchors. Figure SI.12 shows that this molecule is a HOMO-dominated and that is what one would expect due to the fact that the both anchors (*SnMe3* and *SAc*), are HOMO-dominated. The DFT-predicted Fermi energy $E - E_F^{DFT} = 0\ eV$ sits so close to the HOMO resonance because both anchors are pinning in the same direction toward HOMO resonance. Again, it should be noted that both anchors (*SnMe3* and *SAc),* cleave to end up with *Au-C* direct contact (tip side), and *Au-S* contact (substrate side).

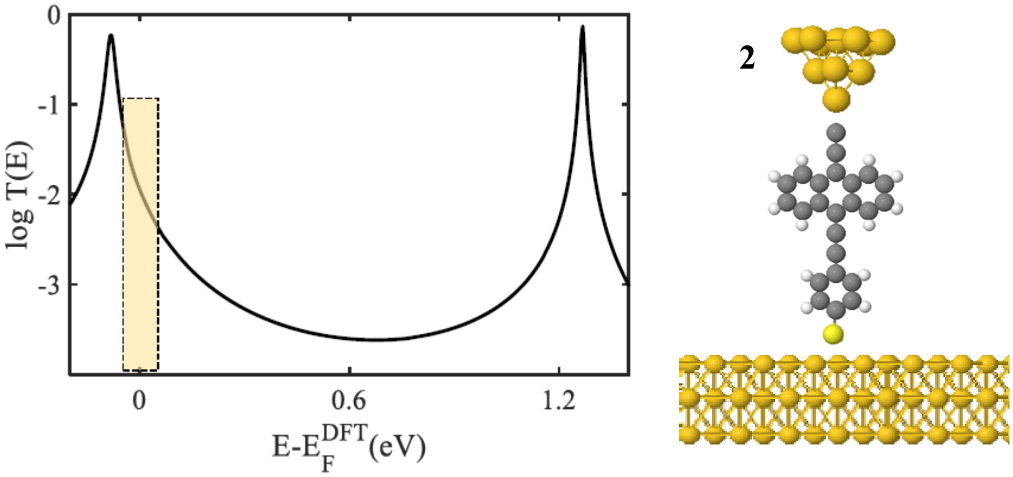

Figure SI.12: Right panel: Schematic illustrations of an asymmetric molecular junction of **2. Left panel:** Zero-bias transmission coefficient $T(E)$ of molecule **2** against electron energy E.

3.3 Case 3: Anthracene-based of *Py* and *SAc* anchor groups:

Case 3 is an asymmetric anthracene with two different anchors including thioacetate and pyridine, as shown in Figure SI.13. As the two anchors are well-known to pin down in an opposite direction, in other words, HOMO- or LUMO-dominated. Furthermore, both anchors are strong so one would expect this molecule to possess a mid-gap Fermi energy ($E - E_F^{DFT} = 0$ eV), rather than a HOMO or LUMO-dominated. Figure SI.13, proves this prediction to be accurate as clearly shown that Fermi energy locates in mid-way between the HOMO and LUMO resonances. Note: The cleavage procedure is happening at the *SAc* anchor and end up with *Au-S* contact.

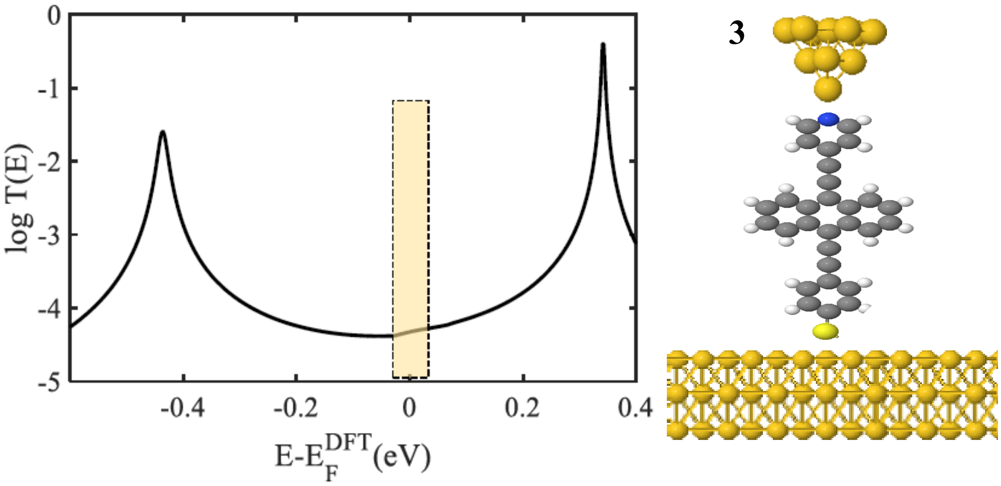

Figure SI.13: Right panel: Schematic illustrations of an asymmetric molecular junction of **3. Left panel:** Zero-bias transmission coefficient $T(E)$ of molecule **3** against electron energy E.

4. Seebeck coefficient *S*

After computing the electronic transmission coefficient for the 3 junctions, we now compute their Seebeck coefficients S. To this end, it is useful to introduce the non-normalised probability distribution $P(E)$ defined by.

$$P(E) = -T(E)\frac{df(E)}{dE} \qquad \text{(S1)}$$

where $f(E)$ is the Fermi function and $T(E)$ is the transmission coefficients, whose moments Li are denoted as follows

$$L_i = \int dE\, P(E)\, (E - E_F)^i \qquad \text{(S2)}$$

where, E_F is the Fermi energy. The Seebeck coefficient, S is then given by

$$S(T) = -\frac{1}{|e|T}\frac{L_1}{L_o} \qquad \text{(S3)}$$

where, e is the electronic charge.

The slope of the transmission coefficient $T(E)$ determines the sign and magnitude of the Seebeck coefficient S. In other words, whether the curve is HOMO or LUMO dominated. Figure SI.14, shows a negative Seebeck coefficient at the DFT-predicted Fermi $E - E_F^{DFT} = 0$ eV and this is due to the fact that molecule **1** is a LUMO-dominated as shown in Figure SI.11 (anthracene of *TMS* and *Py* anchors).

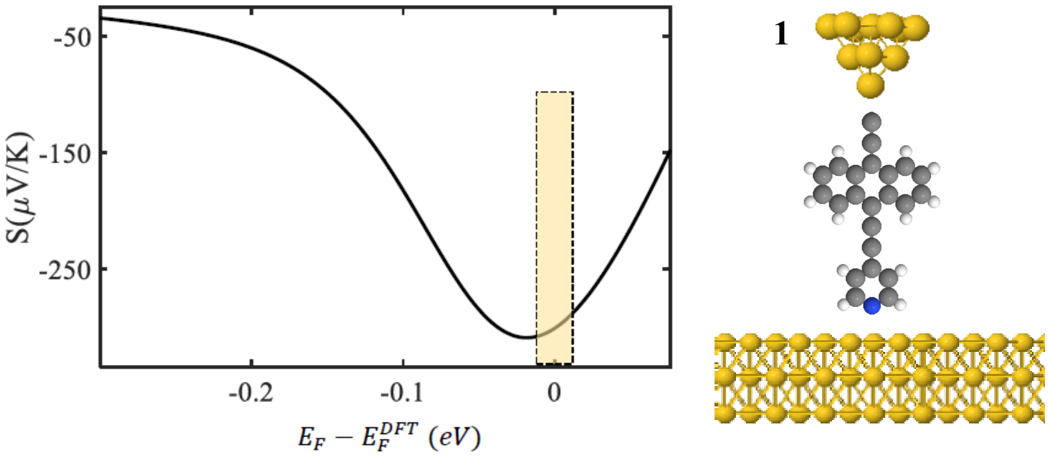

Figure SI.14: Right panel: Schematic illustrations of molecular junction of **1. Left panel:** Seebeck coefficient S of molecule **1** against electron energy E.

In contrast, Figure SI.15 shows a positive S at $E - E_F^{DFT} = 0$ eV, because molecule **2** is a HOMO-dominated molecule as shown in Figure SI.12 (anthracene of *TMS* and *S* anchors). Similarly, Figure SI.16 shows a negative S at $E - E_F^{DFT} = 0$ eV, again because molecule **3** is slightly a LUMO- dominated molecule at the Fermi energy as shown in Figure SI.13 (anthracene of *Py* and *S* anchors).

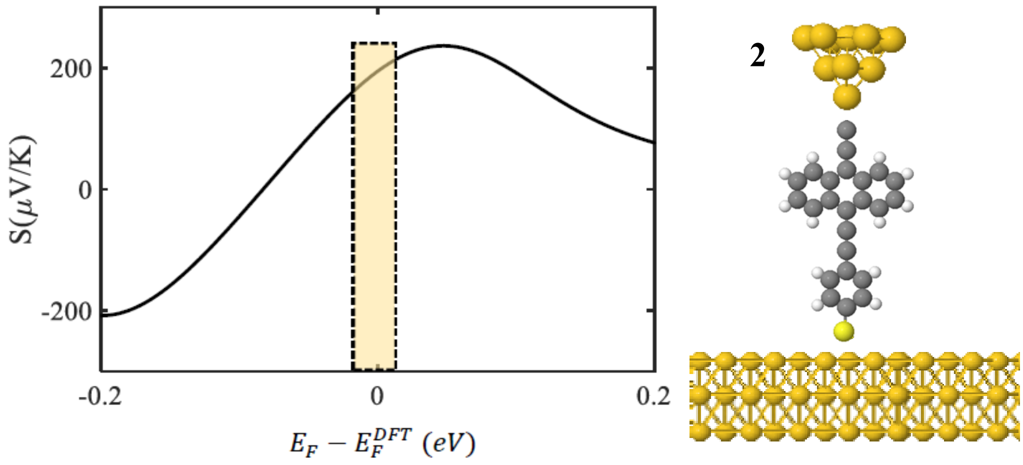

Figure SI.15: Right panel: Schematic illustrations of molecular junction of **2. Left panel:** Seebeck coefficient S of molecule **2** against electron energy E.

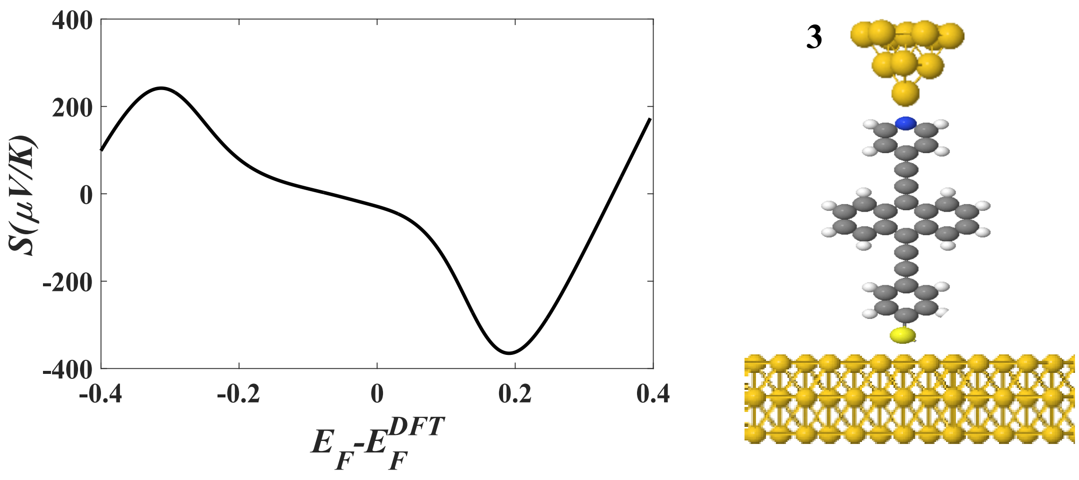

Figure SI.16: Right panel: Schematic illustrations of molecular junction of **3. Left panel:** Seebeck coefficient S of molecule **3** against electron energy E.

5. Flipping characteristic s

Following the simulations of 3 asymmetric anthracene-based molecules in *Au/M/Au* junctions. In this section we shall add an extra segment to the Au/M/Au junction, which is a graphene layer (*Gr*) to form multicomponent *Au/Gr+M/Au*. Experimentally, this means graphene coated gold contact for more detail about the synthetic and STM measurements we guide the reader to[9]. In the present research, the *Gr* sheet is stationary, while the asymmetric molecule flips between two orientations, as shown in Figure 2a of the manuscript. For the flipping purpose we shall investigate 3 scenarios **a**, **b** and **c**.

5.1 Scenarios a:

We employ molecule **1** for this scenario. Figure SI.17 illustrates the components that use to build the flipping junction. It also shows molecule **1** where it consists of spacers and two different anchors groups mainly *SnMe3* and *Py*. Then adding a *Gr* sheet to form the multicomponent compound. Finally, this structure places between two gold electrodes. To achieve the flipping feature, we first link the *Py* anchor to the *Gr* sheet from one end and the *SnMe3* to *Au* substrate from the other end and then place this structure between the *Au* electrodes, as shown in the left panel (orientation-1), of Fig. SI.17. It should be noted the *SnMe3* anchor cleaves when it attaches to the gold metal to form an *Au-C* direct contact. Secondly, we flip molecule **1** so that the *SnMe3* anchor is now attached to the *Gr* sheet, and again place the multicomponent between electrodes as shown the right panel (orientation-1), of Fig. SI.17. We have labelled the two systems as orientation-1 and orientation-2.

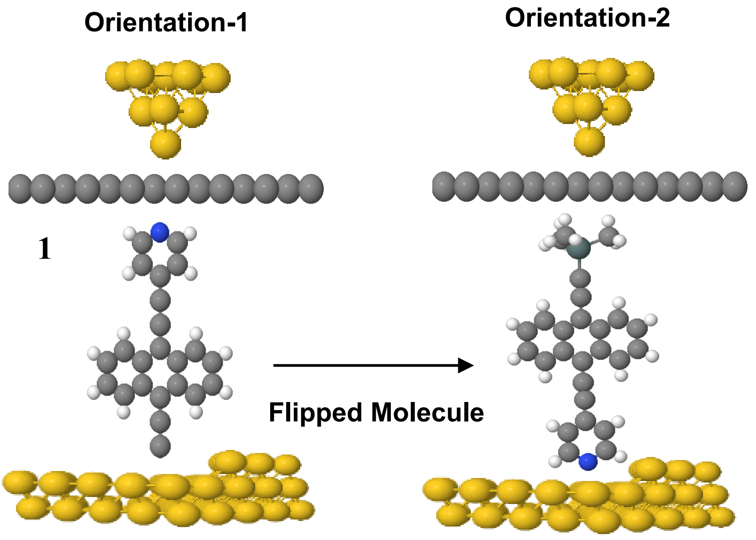

Figure SI.17: Schematic illustration of molecular junctions for two orientations of molecule **1**. Orientation-1 and -2 show how molecule **1** flips between the *Gr* sheet and Au. **Left panel:** Orientation-1 is when the *Py* anchor linked to the *Gr* from one end and the *TMS* to a *Au* from the other end. **Right panel:** Orientation-2 is the opposite, *SnMe3* anchor linked to the *Gr* and *Py* [illegible]

We repeat the same simulations that described in section 3, to calculate the transmission coefficient *T(E)*. Top panel of Fig. SI.18 illustrate the transmission coefficient curves for orientation-1 and -2. This panel proves the two curves to be LUMO dominated. Despite the fact that both curves are a LUMO dominated, however, how far the Fermi energy locates from the LUMO resonance depends on the orientation of molecule **1** between *Gr* and *Au*. In other words, how strong the binding to the *Gr* and *Au* and the type of the anchor. The top panel clearly shows there is a different in the Fermi position when molecule **1** flips from orientation-1 to orientation-2.

Similarly, the Seebeck calculations that described in section 4, apply on the flipping simulations. The lower panel of Fig. SI.18 show the Seebeck coefficient of the two orientations. As the top panel illustrates the two orientations to be LUMO dominated then that should reflect in the Seebeck sign, means both curves possess a negative Seebeck.

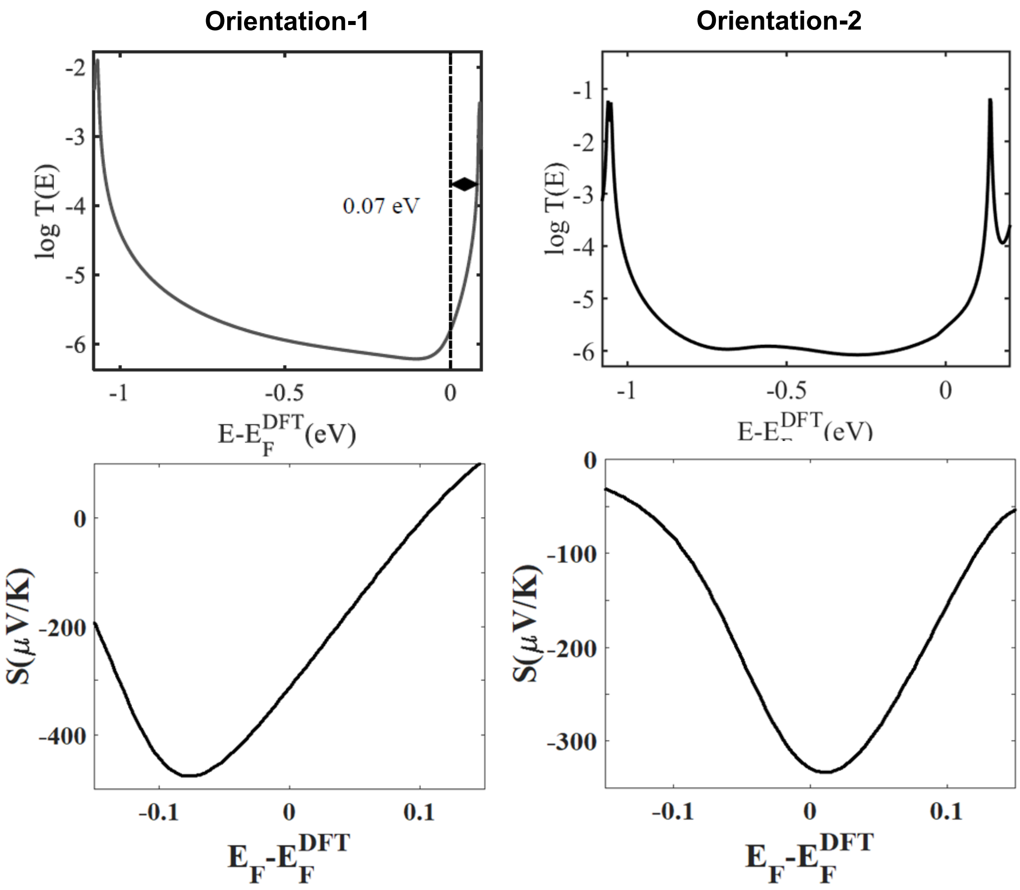

Figure SI.18: Top panel: Zero bias transmission coefficients *T(E)* of molecule **1** against electron energy *E*, of orientation-1 and orientation-2 of Fig. SI.17. The flipping feature shifts the Fermi energy $E - E_F^{DFT} = 0$ from 0.07 to 0.13 eV towards a LUMO resonance (left to right respectively). **Lower panel:** Seebeck coefficients *S* of molecule **1** against electron energy *E,* in two orientations and both exhibit a negative Seebeck.

5.2 Scenarios b:

This scenario employs molecule **2**, and the same procedure that described in scenario a, repeats however with different anchors. Here, we mainly focus on *SnMe3* and *SAc* although these anchors cleave during the flipping procedure. In orientation-1 the *SnMe3* anchor cleaves from the bottom side to form a *Au-C* direct contact as shown in the left panel of Fig. SI.19. Similarly, the *SAc* anchor cleaves from the bottom

side to form a *Au-S* contact as shown in the right panel of Fig. SI.19. Both anchors remain the same when contact the *Gr* sheet.

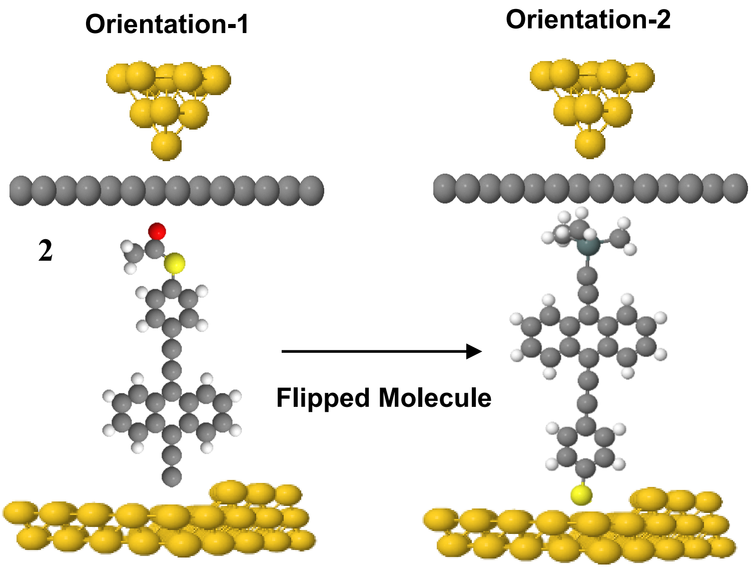

Figure SI.19: Schematic illustration of molecular junctions for two orientations of molecule **2**. Orientation-1 and -2 show how molecule **2** flips between the *Gr* sheet and Au. **Left panel:** Orientation-1 is when the *SAc* anchor linked to the *Gr* from one end and the *TMS* to a *Au* from the other end. **Right panel:** Orientation-2 is the opposite, *SnMe3* anchor linked to the *Gr* and *S* anchor to a *Au* contact.

Top panel of Fig. SI.20 illustrate the transmission coefficient curves for orientation-1 and -2 of molecule **2**. The two curves demonstrate a HOMO dominated curve. Again, although both curves are a HOMO dominated, however, the distance between the Fermi energy and the HOMO resonance determines by the orientation of molecule **2** between *Gr* and *Au*. In other words, how strong the binding to the *Gr* and *Au* and the type of the anchor. It's clearly shown by the top panel, there is a different in the Fermi position when the molecule flips from orientation-1 to orientation-2.

The lower panel displays the Seebeck calculations of molecule **2**. This panel proves *S* of the two orientations to be a positive. This result is expected as the two orientations are a HOMO dominated curves (see top panel).

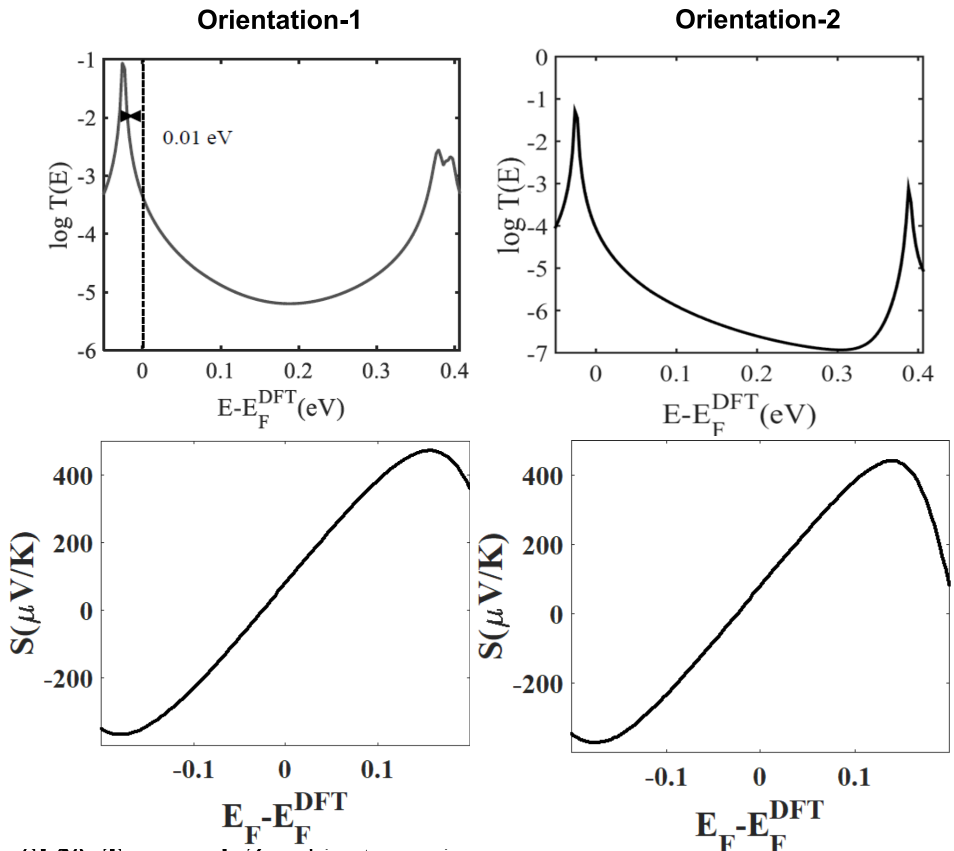

Figure SI.20: Top panel: Zero bias transmission coefficients $T(E)$ of molecule **2** against electron energy E, of orientation-1 and orientation-2 of Fig. SI.19. The flipping feature shifts the Fermi energy $E - E_F^{DFT} = 0$ from 0.01 to 0.15 eV towards a HOMO resonance (left to right respectively). **Lower panel:** Seebeck coefficients S of molecule **2** against electron energy E, in two orientations and both exhibit a positive Seebeck.

5.3 Scenarios c:

Here, we repeat the same procedure that described in scenarios a and b. The difference is the two anchors are *Py* and *SAc,* in this scenario one anchor cleaves during the flipping procedure. Orientation-1 the *SAc* anchor cleaves from the bottom side to form a *Au-S* contact as shown in the left panel of Fig. SI.21. No cleave occurs for orientation-2, where both sides are *Au-Py* and *Gr-SAc* as shown in the right panel of Fig. SI.21.

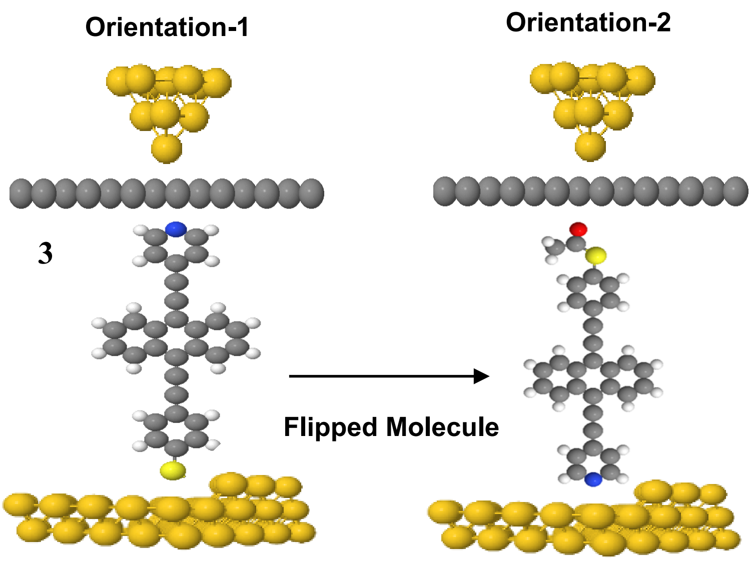

Figure SI.21: Schematic illustration of molecular junctions for two orientations of molecule **3**. Orientation-1 and -2 show how molecule **3** flips between the *Gr* sheet and Au. **Left panel:** Orientation-1 is when the *Py* anchor linked to the *Gr* from one end and the *S* to a *Au* from the other end. **Right panel:** Orientation-2 is the opposite, *SAc* anchor linked to the *Gr* and *Py* anchor to a *Au* contact.

Top panel of Fig. SI.22 illustrate the transmission coefficient curves for orientation-1 and -2 of molecule **3**. The two curves demonstrate an opposite behaviour, meaning a LUMO dominated curve for orientation-1 and a HOMO dominated for orientation-2.

Fig. SI.13 investigates molecule **3** transport in gold-gold junction. It illustrates that molecule **3** possesses a mid-gap transport feature. We attribute that to the conflict between a strong HOMO dominated anchor such as thiol and a strong LUMO dominated anchor such as pyridyl. This is clearly distinguished from molecules **1** and **2,** as **1** is a LUMO dominated (see Fig. SI.11), and **2** is a HOMO dominated (see Fig. SI.12), even though both molecules are asymmetric. This finding strongly suggests that one of the anchors overcomes the other, for example, for **1** *Py* > *TMS* and **2** *SH* > *TMS,* therefore, there is either a HOMO or LUMO trend, but not mid-gap likewise **3**.

Now, one would argue that the thiol anchor is stronger than pyridyl in molecule **3** and therefore should obtain a HOMO domination than a mid-gap. To satisfy this concern, there are many studies[10-12] demonstrate the pyridyl anchor is much stronger on a rough Au substrate, thus, we use an ad-atom in our simulations. The second supporting point for this concern is also an experimental evidence (XPS measurements), the percentage of the two orientations as it shall be discussed in the following section.

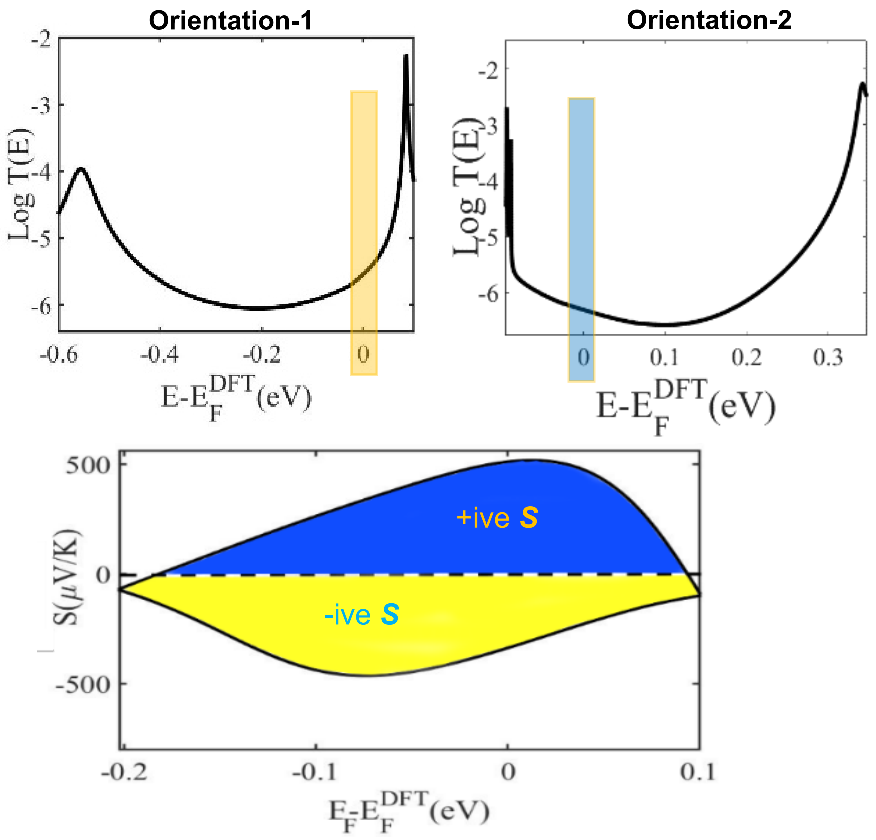

Figure SI.22: Top panel: Zero bias transmission coefficients *T(E)* of molecule **3** against electron energy *E*, of orientation-1 and orientation-2 of Fig. SI.21. The flipping feature switches the Fermi energy $E - E_F^{DFT} = 0$ position from LUMO to HOMO (left to right respectively). **Lower panel:** Seebeck coefficients *S* of molecule **3** against electron energy *E,* in two orientations and the Seebeck coefficient switches from a negative to positive sign (yellow to blue respectively).

6. References

1. J. M. Soler, E. Artacho, J. D. Gale, A. García, J. Junquera, P. Ordejón and D. J. J. o. P. C. M. Sánchez-Portal, *J. Phys.: Condens. Matter*, 2002, **14**, 2745.
2. A. Emilio, E. Anglada, O. Diéguez, J. D. Gale, A. García, J. Junquera, R. M. Martin, P. Ordejón, J. M. Pruneda, D. Sánchez-Portal and J. M. Soler, *J. Phys.: Condens. Matter*, 2008, **20**, 064208.
3. N. Kobko and J. Dannenberg, *The Journal of Physical Chemistry A*, 2001, **105**, 1944-1950.
4. C. D. Sherrill, *School of Chemistry and Biochemistry, Georgia Institute of Technology*, 2010.
5. W. Chen, J. R. Widawsky, H. Vázquez, S. T. Schneebeli, M. S. Hybertsen, R. Breslow and L. Venkataraman, *Journal of the American Chemical Society*, 2011, **133**, 17160-17163.
6. A. K. Ismael and C. J. Lambert, *J. Mater. Chem. C,* , 2019, **7**, 6578-6581.
7. C. Wang, A. S. Batsanov, M. R. Bryce, S. Martin, R. J. Nichols, S. J. Higgins, V. M. Garcia-Suarez and C. J. Lambert, *Journal of the American Chemical Society*, 2009, **131**, 15647-15654.
8. V. Obersteiner, D. A. Egger and E. Zojer, *The Journal of Physical Chemistry C*, 2015, **119**, 21198-21208.
9. T. L. Bennett, M. Alshammari, S. Au-Yong, A. Almutlg, X. Wang, L. A. Wilkinson, T. Albrecht, S. P. Jarvis, L. F. Cohen and A. Ismael, *Chem. Sci.*, 2022, **13**, 5176-5185.
10. S. M. Hou, J. X. Zhang, R. Li, J. Ning, R. S. Han, Z. Y. Shen, X. Y. Zhao, Z. Q. Xue and Q. Wu, *Nanotechnology*, 2005, **16**, 239-244.
11. S. Y. Quek, M. Kamenetska, M. L. Steigerwald, H. J. Choi, S. G. Louie, M. S. Hybertsen, J. Neaton and L. Venkataraman, *Nat Nanotechnol*, 2009, **4**, 230-234.
12. R. Stadler, K. S. Thygesen and K. W. Jacobsen, *Physical Review B*, 2005, **72**, 241401.

13- X. Wang_et al. "Tuning Fermi Level Alignment in Large Scale Self-Assembled Oligo (phenylene-ethynylene) Derivatives". ***Nanoscale Horizons.*** (submitted)
